# Water supply of ancient Egyptian settlements: the role of the state. Overview of a relatively equitable scheme from the Old to New Kingdom (ca. 2543–1077 BC)

**DOI:** 10.1007/s12685-015-0150-x

**Published:** 2016-01-12

**Authors:** Delphine Driaux

**Affiliations:** McDonald Institute for Archaeological Research, University of Cambridge, Cambridge, England UK; UMR 8167, Orient et Méditerrannée (Équipe Mondes Pharaoniques), Université Paris-Sorbonne (Paris IV), 1 rue Victor Cousin, 75230 Paris Cedex 05, France

**Keywords:** Ancient Egypt, Water supply, State, Local administration, Settlements

## Abstract

The study of the textual and archaeological evidence shows that the water supply of the settlements of ancient Egypt seems to have worked on a simple and a relatively equitable scheme, at least from the Old Kingdom until the New Kingdom (ca. 2543–1077). The water supply of the inhabitants was completely managed by the state, through the local administration which was charged to bring the water, in general from a rural area, into towns and cities and to redistribute it to the inhabitants. The method of supply is illustrated by several sources of evidence, in particular by the well known case of the “water-carriers” of the village of Deir el-Medina. Thus, drawing together text and archaeology, this paper will demonstrate that over an extended period, even when the city was far from a water source, the state did not set up complex installations such as pipe networks or wells to bring water, but preferred a simpler system using the manpower available.

## Introduction

From a historical point of view, the control of water, and in particular the control of the flood, has played an important part in the development of the “hydraulic civilisations” to which Egypt belongs (Butzer 1976). The scene pictured on the mace-head of King Scorpion,^1^ showing the king holding a hoe and digging a canal, demonstrates that at the end of the Predynastic era, irrigation was not natural any more but already modified or even artificial (Butzer 1976, pp. 36–37). If the power of the king came from his warlike qualities and hunter’s prowess (Bonhême and Forgeau 1988, p. 163), this representation on such a symbolic object suggests also a strong connection between power and water. As D. Bonneau (1982, p. 80) pointed out, the control of water is one of the constant pillars on which is based the political system of ancient Egypt. Beyond his management of irrigation, directly linked to his nourishing function, the king is also responsible to the gods for the regularity of the flood (Bonneau 1982, p. 69; Gautier and Midant-Reynes 1995, pp. 113–122). By claiming to be the guarantor of a regular and fertile flood, and annihilating its destructive effect, the king appropriated for himself the sacred character the flood, but also at the same time, the sacred character of the water (Grimal 1994, p. 195).

It was the King who laid down rules about irrigation but also about water usage in general (Bonneau 1982, pp. 69–71; Goyon 1982, pp. 61–63). However, written sources, like the text called *Duties of the Vizier* (Van den Boorn 1988), establish that all the kinds of works related to water were entrusted to the Vizier—second in power only to the king. Therefore, the water control was one of several important factors that contributed to the emergence of the ruling class and bureaucracy (Goyon 1982, pp. 63–65; Van den Boorn 1988, pp. 234, 240–242).

While many studies have been carried out on water management in ancient Egypt in terms of the administration of irrigation, the role played by Pharaonic institutions in everyday water management is not very well known. Yet, as this paper will show,^2^ considerable textual and archaeological evidence exists to show that, in settlements, a consistent system was in place, working on a simple and relatively equitable scheme, from at least the Old Kingdom (second half of the 3rd millennium) through the New Kingdom (ca. 1539–1077 BC). During this period, it seems that the state, through the local administration, acted directly in terms of controlling the water supply of its inhabitants.

## Pyramid towns

One of the earliest pieces of evidence is a limestone stela engraved with the Decree of Pepi I (ca. 2276–2228 BC) which mentions the location and the kind of installations where water was drawn for the pyramid towns of Sneferu at Dahshur:^3^

*jw wḏ.n ḥm(.j):*

*nfr r jp mrw šw šdwt ẖnwt nhwt*

*m njwty jptn*

*(My) Majesty has decreed:*

[11] *That it is forbidden to reckon* (with a view to taxing them) *the canals, the pools, the šdt*-*wells, the ẖnmt*-*wells and treesfrom these two pyramid towns.*^4^

The pyramid towns were founded by the state for specific purposes, and in particular housed the priests attached to the royal funerary cult. In general, these towns were built relatively close to the king’s pyramid, at the edge of the desert, and enclosed by a wall.^5^ Sneferu’s pyramid towns in Dahshur, mentioned in the text above, have never been excavated. However, the terms related to the water installations^6^ used here seem to indicate that the latter were not located within the settlements themselves. Based on what we know of other pyramid towns, they were probably outside the settlement, on the agricultural estates attached to these towns and so belonging to them as institutions (Moreno Garcia 2008). Indeed, these estates were generally subjected to taxes, relating particularly to agricultural yields (Goedicke 1994, pp. 191–194). Whilst the latter required water, the water itself did not fall under any form of taxation, and especially not the water used for sustaining a population working for the state.

## “Watering places” and “drinking places”

On a Middle Kingdom limestone stela (No. 1774) preserved in the Museo Archeologico di Firenze (Varille 1935–1938; Fischer 1972), dating approximately from the reign of Senusret I (1962–1928 BC), a man named Mentuweser enumerates his personal wealth but also the acts done for his city:

*… jr.n.j swrt n njwt(.j)*

*… I made a watering*-*place for (my) city*.

**Fig. 1:**
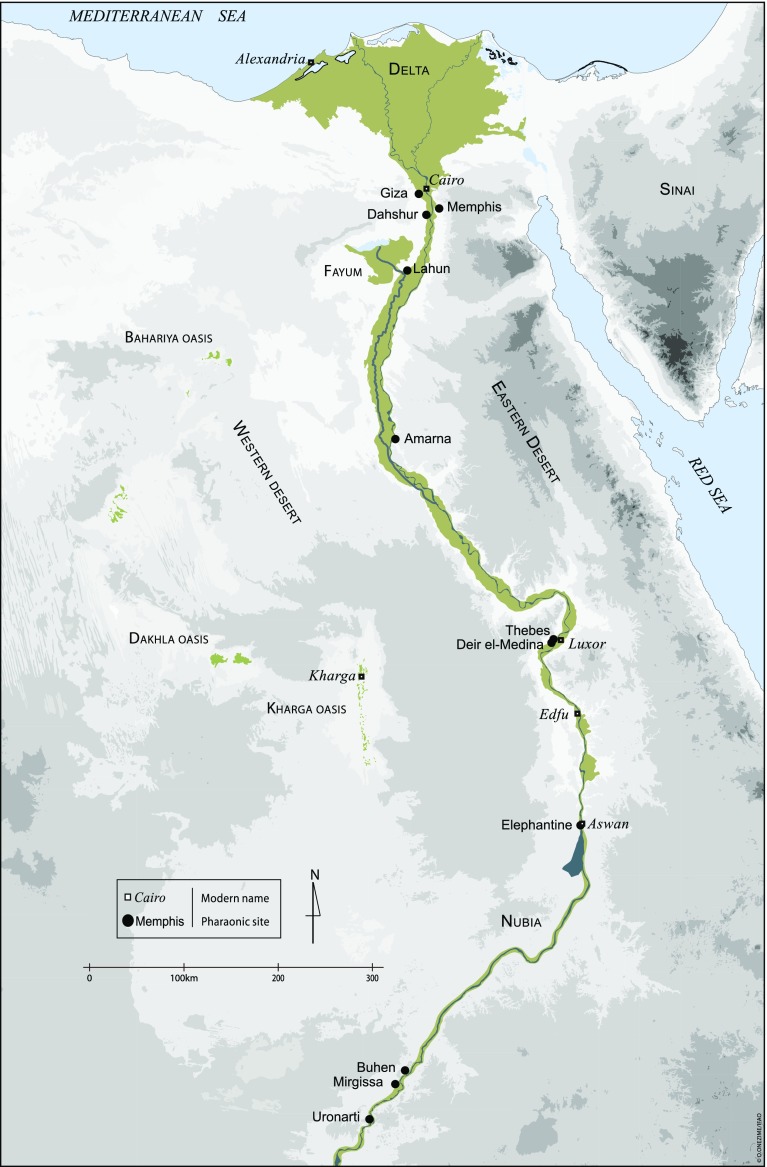
Map of Egypt (O. Onézime–Ifao)

Mentuweser did not mention the name of the town, but we can assume that he was a local dignitary in the Theban area, because, according to his titles, he was in charge of the dining quarters and kitchen of Montu’s temple, a god usually worshipped in Thebes and its surrounding area (Fig. 1). This kind of text—where officials speak highly of their success and career highlights—is quite common in this period, and this stela is one of the first to mention the part played by an official in the water supply of a city.

Contemporary with this monument, a sandstone stela (Aswan Museum No. 1373) discovered in Elephantine provides more information (see Habachi 1985, pp. 36–37, Fig. 3, pl. 24; Franke 1994, pp. 154–175). Here, Sarenput I, mayor of the town^7^ during the reign of Senusret I, reviews all the work done for the city and says:

*… jrj.n.(j) ʿt n wʿb st swr n ȝbw*

…* (I) made a house for the priest(s), and a drinking-place for Elephantine*.

**Fig. 2:**
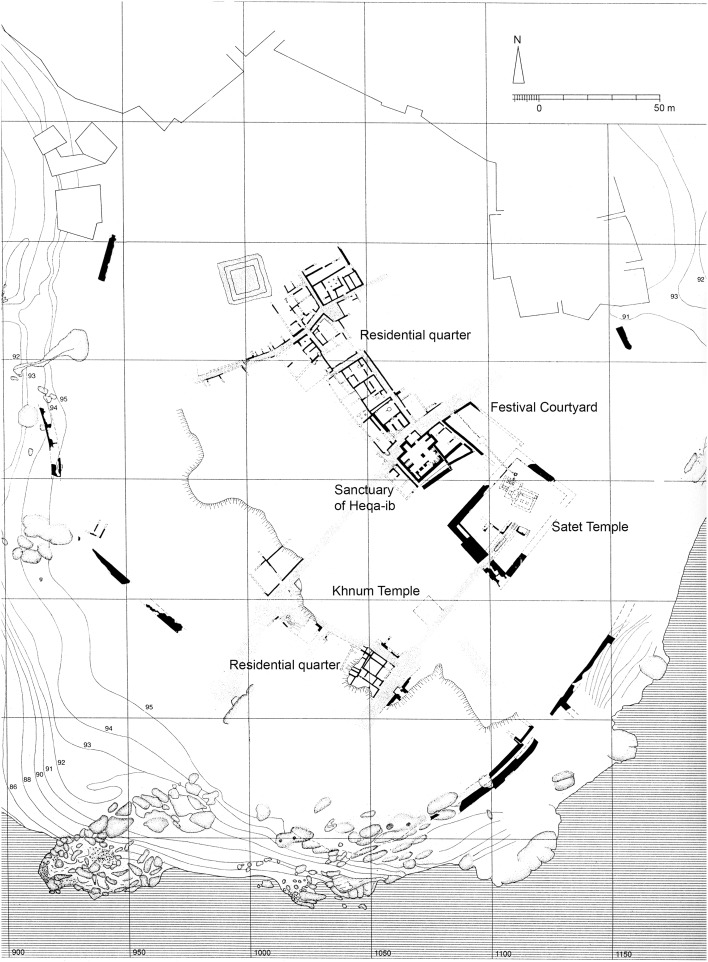
Elephantine during the Middle Kingdom and Second Intermediate Period (after Von Pilgrim 2010, Fig. 1)

We have no direct evidence for the precise location of this “drinking-place”, although some hypotheses have been made, and given the location of the town on an island in the middle of the Nile, surrounded by an enclosure wall, it seems reasonable to assume that the drinking-place mentioned was located inside the town itself. C. Von Pilgrim has suggested it was situated in a large courtyard northwest of the Satet Temple (Kaiser et al. 1997, pp. 152–157) (Fig. 2). Equipped with a sunken limestone basin, this courtyard would have been used for ritual purposes, and in particular the arrival of the flood. This place is easily accessible, in the centre of the settlement, yet it is, however, hard to imagine that a place primarily serving cultic functions, and thus sacred, was used on a daily basis to supply the population of Elephantine with water.

**Fig. 3:**
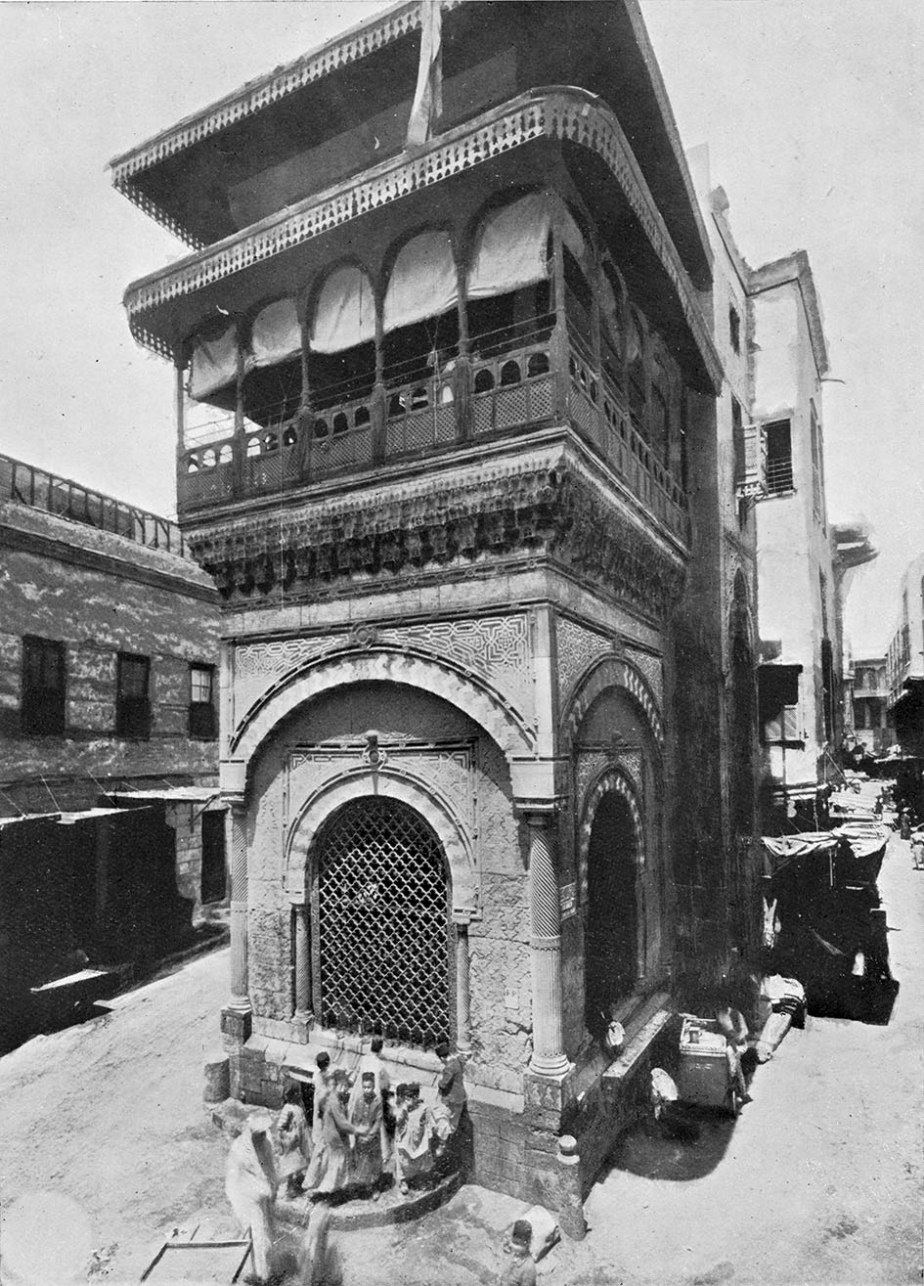
Sabil of ʿAbd ar-Rahman Katkhuda – Cairo (Pauty 1936, pl. XIII © Ifao)

In neither case does the vocabulary used (*swr*; *swrt– Wb* III, 429, 4)^8^ imply any particular kind of technical water installation, such as a well, for which we have specific words in Egyptian hieroglyphic—the two more common words being: *ẖnmt* (*Wb* III, 382, 10–15) and * šdt* (*Wb* IV, 567, 1–2.).^9^ In the case of Elephantine, the water had to be brought from the Nile to the city, probably by water-carriers working for the community, and directly dependent on the administration, as outlined below. As a result, these “drinking-places” were probably installations equipped with jars and located in small squares, perhaps in the middle of a town, or at different points throughout a settlement. The water would have been replenished every day, and the inhabitants permitted to take the quantity of water they needed—maybe under the control of a scribe. If this were the case, then a strong parallel exists between them and the drinking-places and water-tanks called *sabil* in modern Cairo (Fig. 3). These cisterns, built throughout the city since the 16th century by rich private individuals as pious foundations (*waqf*), provided the inhabitants of the city with free water until the second half of the 19th century (for the *sabil*, see Pauty 1936, pp. 22–30; Raymond 1979).

## Evidence from New Kingdom workmen’s villages

While for the Pharaonic period we cannot connect with any certainty the drinking-places mentioned in texts with archaeological evidence, discoveries at two workmen’s villages allow for some insight on this topic.

**Fig. 4:**
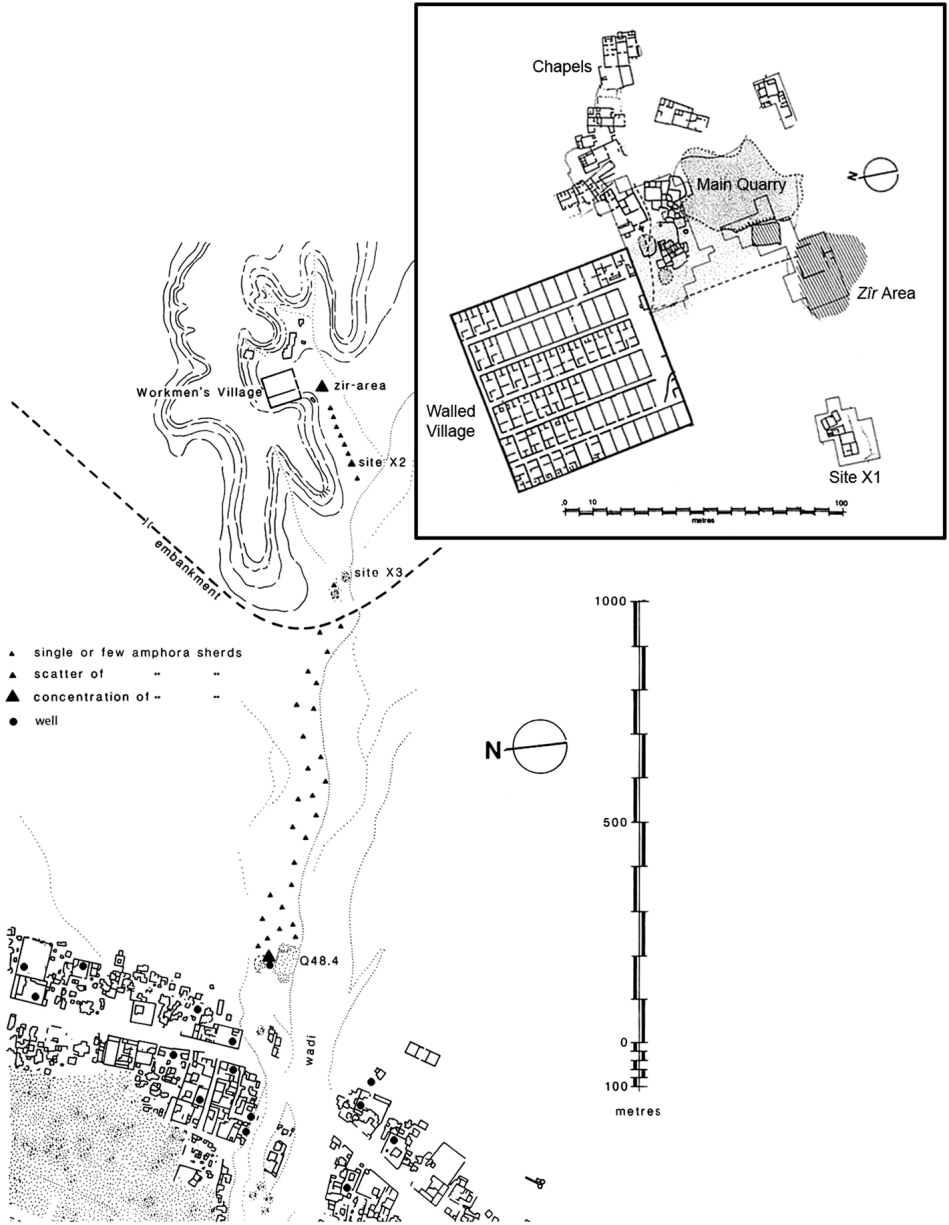
Map showing the edge of the main city of Amarna and the workmen’s village (after Kemp 1984, Fig. 1.3; Rose 1987, Fig. 9.6 © EES)

The first site is the New Kingdom workmen’s village at Amarna, situated around 1.2 km east of the main city, at the edge of the desert.^10^ This isolated village was created by the royal administration, probably for the workmen engaged in royal tomb construction, and at some stage perhaps used for the policing of the desert (Kemp 1987, pp. 43–49). Surrounded by a mud-brick wall approximately 69 m^2^, the village contains 72 houses, built along similar and simple plans, arranged along a series of parallel and narrow streets (Fig. 4). Except for a large house in the southeast corner, presumably for an official in charge of the community, the houses were built according to a strict planning framework that allowed for little extra space inside the enclosure wall. There was thus no space inside the village for any other kind of buildings such as chapels or installations related to the provision of foodstuffs or water. There was certainly no space for a well, such an installation in any case being almost impossible to establish in this desert area, since the water table is too deep (Kemp 1984, p. 6).

**Fig. 5:**
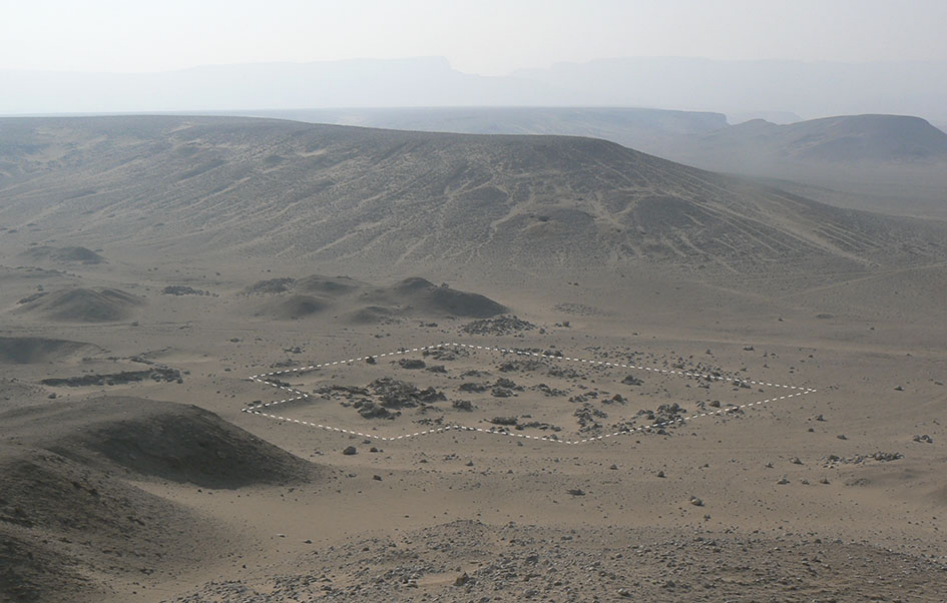
View of the *zîr*-area, looking south-east (© D. Driaux)

The easiest solution was to bring water to the village. Archaeological evidence—namely, scatters of sherds from vessels thought to have served water transport—on the route leading to the workmen’s village and on the ground in front of it (Kemp 1984, pp. 60–80; Renfrew 1987), show that a water delivery system existed to meet the needs of both the inhabitants of the village and also the animals they tended. Due to the status of the settlement—made by the state, for its own purposes—it makes sense that this isolated village, with few opportunities for self-sufficiency, was supplied by the administration. The origin of the water itself seems to have been one of several wells located at the edge of the main city (Fig. 4). The excavation of one well revealed that it belonged to an administrative centre (Q. 48.4), delimited by a rectangular enclosure wall and housing several workshops producing ceramics, faience jewellery, and glass objects, organized around a central open space (Kemp 1989, pp. 15–55). Termed “intermediate centres” by B.J. Kemp (1989, pp. 56–63), this kind of building was not attached to formal institutional workshops and was not under the immediate control of the temples and palaces. However, they seem to have been administered by a local official on behalf of the king. The state therefore delegated responsibility to these establishments for sending goods, and the water from their wells to the workmen’s village. Potsherds found along the route to the village, but also in the vicinity of the well cited above, show that the water was carried in a particular kind of vessel: imported and reused Canaanite amphorae or imitations of this form (Renfrew 1987, pp. 94–98; Rose 1987, pp. 124–126). These handled jars have a slender form (Rose 2007, pp. 147–49, 292–294), a size (between 50 and 60 cm high) and a capacity (ca. 19 l) which allow a relatively easy transport, in particular on the back of a donkey. The water was then carried to a point some distance from the entrance of the walled village, marked by a boundary line, where it was unloaded and brought a few meters further to a spot in front of the village, named by excavators as the “*zîr*-area” because of the large number of fragments of water jars (Arabic *zîr*) found there (Fig. 5).^11^ Interpreted as “an interchange place” by B.J. Kemp, this area, delineated by a low enclosure wall, seems to have been set up to discourage the water-carriers from approaching the village directly (Kemp 2012, p. 194). The water was then poured into the *zîrs* which, with their rounded bases, stood in small emplacements built of stone, brick rubble and marl mortar. The water jars provided a standing source of water and villagers then took the water they needed inside the village. Without any written documents, it is quite difficult to estimate the daily needs of the inhabitants; one *zîr* contained ca. 35 l but this was probably not enough to cover all daily needs, whether for a single man or a family. If that is so, then the jars would have needed to have been refilled more than once. The number of water jars contained in this area is estimated at around 50. This number is also the estimated number of houses, on average, occupied here at any one time (Kemp 1984, p. 80). On the basis of one resupply of water stocks per day, almost 1750 l of water had to be sent to the village daily (35 l × 50 *zîrs*). A simple calculation gives 92 as the number of Canaanite amphorae necessary to bring this important supply of water; and if we consider that one donkey was able to carry two amphorae, one on each side, then 46 donkey journeys were required. Through such calculations we can build a general picture of the logistics of supplying the Amarna workmen’s village with water. Yet many questions remain: we do not know precisely how the supply was organized, how many water-carriers were involved, or how many journeys from the city to the village were undertaken during one day. Nevertheless, due to the particular character of the settlement, a very simple approach to water management is suggested by the remaining evidence, and one without any waste, since the demand matches the supply. Finally, the presence of the *zîr*-area outside the walled village, but functioning with it, shows that it was included in a larger urban plan. With this in mind, the *zîr*-area could be related to the “drinking-places” mentioned in textual sources.

**Fig. 6:**
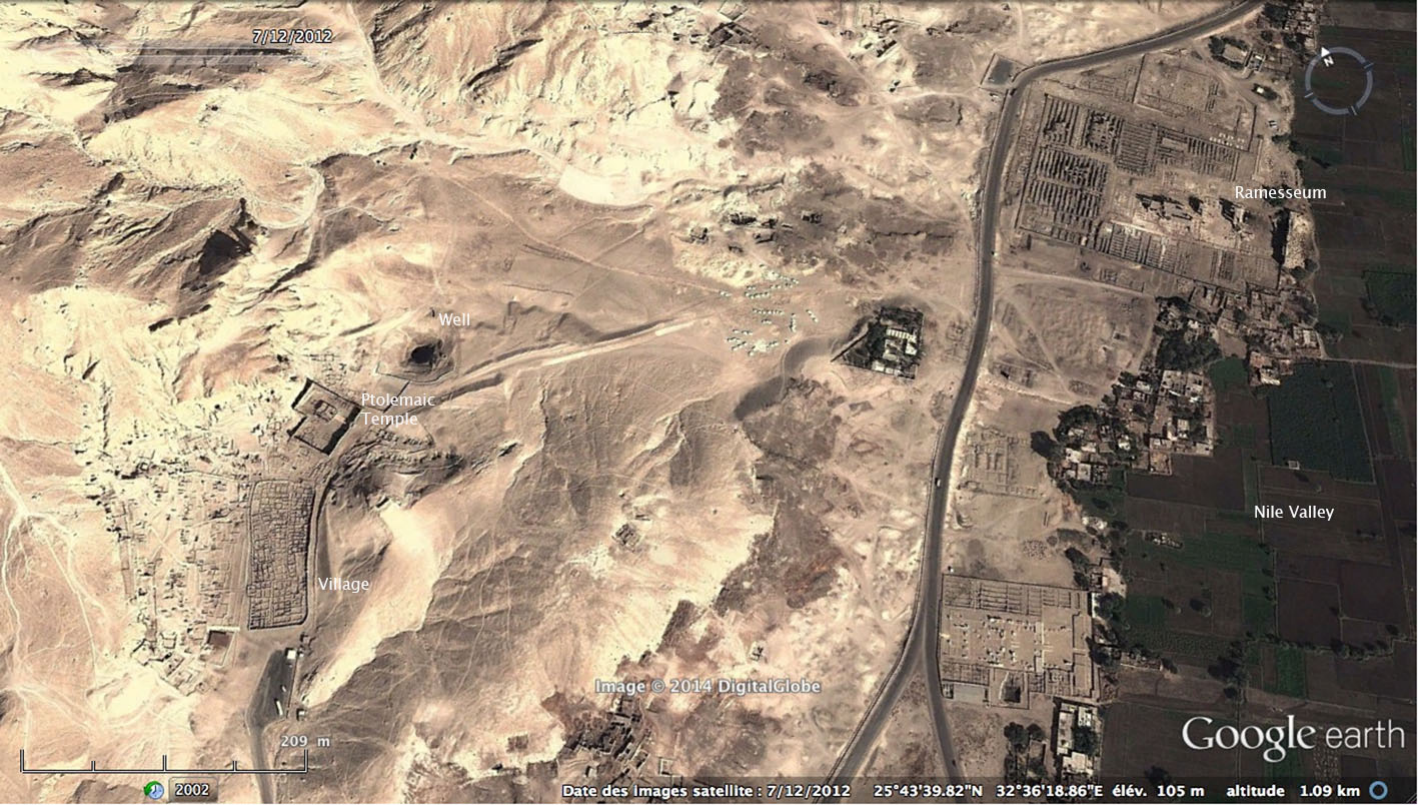
View of Deir el-Medina and its environs (Google Earth © DigitalGlobe)

The second workmen’s village shows the same kind of organization. Situated at the boundary with the desert, the small village of Deir el-Medina at Thebes housed the workmen in charge of the construction and decoration of the royal tombs in the Valley of the Kings for almost five centuries (ca. 1500–1069 BC. See McDowell 1999, pp. 18–23). Occupying an area of ca. 5600 m^2^, the village, enclosed by a mud-brick wall (Fig. 6), contained almost 68 houses in its final phase (Valbelle 1985, pp. 114–116).^12^ Like Amarna, Deir el-Medina was under the control of the royal administration; the village and its inhabitants belong to a specific and large-scale institution generally referred to as “The Tomb”^13^ in the administrative documents. Placed under the authority of the Vizier, the isolated inhabitants were supplied with tools, materials, food and water from economic centres in the Nile Valley. Unlike Amarna, there is little archaeological evidence for the logistics of the village’s water supply; however, the site has yielded important written documentation (mainly on ostraca) containing a lot of relevant information. The two chiefs of workmen as well as the scribe (who together managed the community)^14^ define their own duties in the textual sources; one of these duties was the supply of water. Another special category of personnel, attached to the community of villagers and called *smdt*,  is also mentioned. They are employed by “the Tomb” for specific activities, and, in particular, for various supply tasks (Cerný 2001, pp. 183–190). The men in charge of water supply form a particular subgroup of workers and have a specific title: *jnw*-*mw*, “the one who brings the water” that we can translate as “water-carrier”.^15^ According to the documentation, they belong to the lower class of society, live outside the village and had to hire, from the workmen, the donkeys used to carry the water (Eichler 1991). Unfortunately, we do not know exactly from where they were drawing the water. We can assume, however, that they had to make several round-trips to plots of land that were the property of “the Tomb” or the royal institutions (Valbelle 1985, pp. 88–90, 144–147) in the flood plain, to access the nearest canal or well. The water was then brought up to the entrance of the settlement where the guard in charge of the watch took delivery of it, before it was apparently transferred into a kind of cistern in ceramic situated in a square just outside the gate of the village.^16^ As at Amarna, the villagers of Deir el-Medina had to come outside to get their daily supply, possibly under the control of a scribe who noted each quantity taken, since there was no personal *zîr* for each household but only one large communal cistern.

For both villages, the fundamental role of the state in water supply via the local administration is quite clear. The state took charge of the water supply from beginning to end, choosing simple methods such as manpower, often available in large quantities and using their own estates and facilities.

## Water supply at fortresses

This simple system chosen by the administration could be explained, at some point, by a lack of technology and/or knowledge. Although the Egyptians were able to build wells in the flood plain, they do not seem to have had the technical ability or desire to transport water further afield without carrying it directly, at least during the period under consideration. Evidence from fortresses, also founded by the state, but this time for military purposes, supports this assertion.

**Fig. 7:**
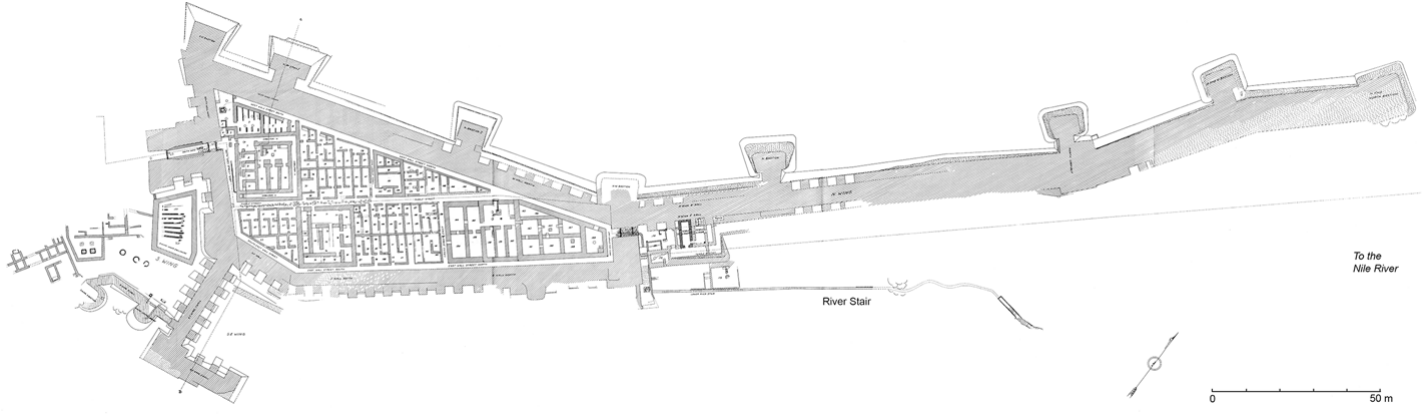
Map of the fortress of Uronarti (Dunham 1967, map III © Museum of Fine Arts, Boston)

The Middle Kingdom fortresses of the Second Cataract were built to control the frontier between Egypt and Nubia.^17^ Some of these settlements were situated on rocky granite outcrops, which did not allow for wells to be dug. With no possibility of raising the ground water, the best solution was to go to the Nile. In the case of the fortress of Uronarti, for instance, the response was once again to organize a delivery system, using a long staircase (240 m) cut into the rock (Fig. 7), and leading from the eastern gate of the fortress to the river (Dunham 1967, pp. 19–20). Uronarti offers, in a way, many of the same conditions found at the workmen’s villages: an enclosed settlement, under the control of the administration, where state employees live together relatively far from a water source. Here, there is no evidence for water-carriers like at Deir el-Medina, but one might imagine that the water supply was one of the tasks undertaken by members of the garrison themselves.

## Desert expeditions and their influence

These simple solutions for supplying water to difficult areas leads to a final case study: that of expeditions sent to the desert to reach mines and quarries, often far from the Nile Valley. Such expeditions sometimes had to walk up to several hundred kilometres in the desert, under the hot sun. The lack of water was thus a serious threat and the distribution of water-supply points on the road was essential (Gasse 1994). Water management was here still a duty of the state, but in this case it was impossible to set up a water-carrier system. In these remote areas, the administration made the choice to use cisterns and wells, technically quite simple at the beginning, but increasingly elaborate with time.^18^

Several texts mention how the Pharaoh “has dug himself” or “has ordered the digging” of some wells. Among them, the Kanais inscription tells us how during a survey of the eastern desert, King Seti I (1290–1279 BC) was surprised at the lack of water along the road leading to the mines and how, after looking for a better place to dig a well, he brought specialized workmen to do it^19^:

1. *… hrw pn jst ḥm.f smȝʿ.f ḫȝswt r r-ʿ ḏww Ȝb.n*
2. *ib.f mȝ ẖȝwt innw ḏʿm jm.sn jr m-ḫt ḥm.f m ṯsy m rḫw jtryw ʿšȝw jrt.jn.f sḫny ḥr wȝt r wȝwȝ sḥ ḥnʿ jb.f ḏd.jn.f ḳsn.wy wȝt jwty mw.s ḫpr mi m-ʿ m-ʿ*
3. *mšȝw sswn r.f jrf nḏȝ ḫḫ.sn jn-m ʿḫm.f jbt.sn tȝ wȝww ḫȝst wsḫ.t(j) jʿnw n.f s jby ḥr jȝtw js m-‘*
4. *nḏ.j ḫrt.sn, jry.j n.sn ʿ n sʿnḫ.sn …*
5. *… jr m-ḫt ḏdw ḥm.f nn mdw.f ḫr jb.f ḏs.f, dbnbn.in.f ḥr ḫȝst ḥr wḫȝ st irt ẖnwt … sḥnw kȝwtyw*
6. *m jnr r šdt ẖnmt ḥr ḏww n*-*mrwt sṯs.f wrdw sḳbb.f jb nwḫ m šmw.*
7. … *On this day, now His Majesty surveyed the deserts right up to the mountains. (In) his mind,*
8. *he desired to see the mines from which electrum is brought. When His Majesty had gone many miles, then he made a halt upon the road to think things over. Then he said: “How difficult is (such) a waterless road! What happens to*
9. *travellers, then, to relieve their parched throats? Who shall quench their thirst? The (inhabited) land is far away and the desert is (so) wide–woe betide the man who thirsts in the wilderness! How*
10. *can I care for them, so that I (can) provide a lifeline for them ….*
11. *… After His Majesty had formulated these matters in his own mind, he reconnoitred he desert, seeking out a (suitable) place for making a well*- *ẖnwt ….*
12. *Stoneworkers were commanded to dig a well-ẖnmt in the mountains, in order that he might uplift the weary and cool the heat of him who was parched by the summer heat.*^20^

Whilst these documents testify often to strong political interest in managing territory, they also show an efficient administration able to address different kinds of problems, particularly those concerning water supply. What is more, the installation of wells in such contexts probably helped develop a progressively better knowledge about the digging of wells, which was imported into the Valley and some settlements.

**Fig. 8:**
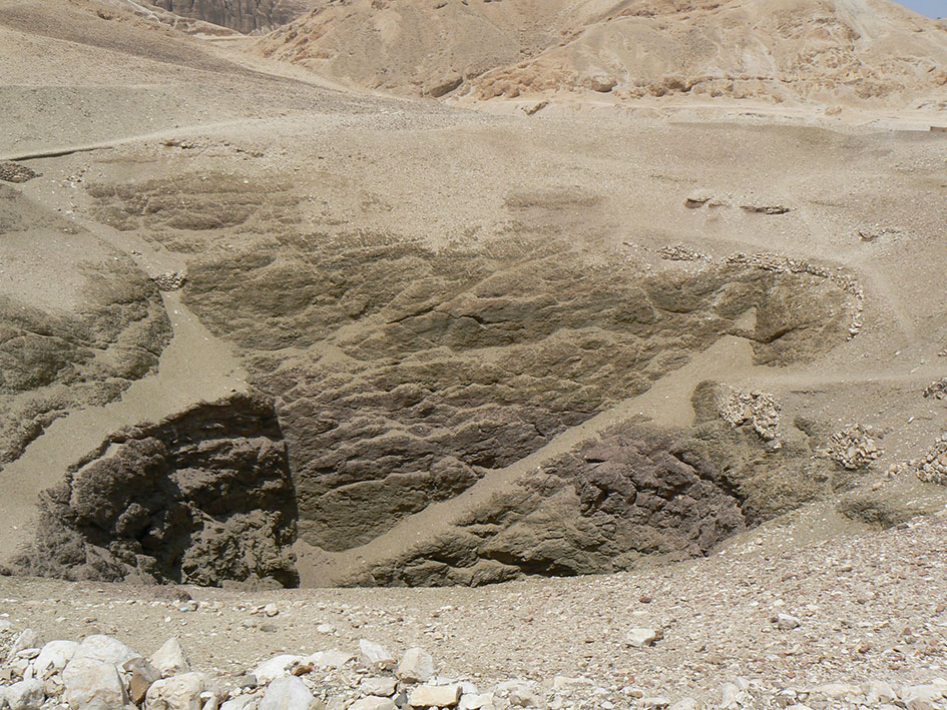
Mouth of the well of Deir el-Medina, looking north (© D. Driaux)

This phenomenon can be observed at Deir el-Medina. Whilst the water supply system set up seems to have worked efficiently, about one century before the end of the settlement, during the reign of Ramesses III (1187–1157 BC), the administration records on an ostracon the first attempt to dig a well, probably ordered by the state itself, in the vicinity of the workmen’s village. Situated a few hundred metres north of the settlement, in the desert (Fig. 6), this large well extended 52 m down to the water level (Fig. 8). Although it is usually believed that the well never functioned (McDowell 1999, p. 18; Morris 2005, p. 38 and more recently Eyre 2009, p. 115), a reassessment of the evidence suggests that it did (Driaux 2011). Despite the importance of the project, we do not know if the quantity of water drawn from the well was sufficient to cover the needs of the villagers or whether it worked for a long period of time or simply dried up quickly. The only thing we know is that, even during this time, the administrative documents still continued to mention water-carriers since, even with a well, such men are still necessary. Officials were likely aware of the difficulties of the water-carriers’ work but alleviating their burden is unlikely to have been the main reason for digging the well. Instead, the initiative might have been prompted by changes within the village, like an increase in population or problems with water delivery.^21^ By digging a well while keeping the water-carrier system, probably making the system more effective, the state seems to have adapted its methods in response to new situations and needs.

## Closing remarks: change and continuity

It appears that the use of a well near the village of Deir el-Medina, constructed with new techniques, does not seem to have changed completely the system of water supply initially set up by the administration, a model that seems to have worked perfectly for many generations. There is a simple and logical basis to this—with settlements founded by the state to house a specific population working for Pharaoh, the state, through the local administration, was in charge of supplying their inhabitants. The administration tended to use simple methods for getting supplies from its estates, using the manpower available to deliver it. It is no surprise, therefore, that the state chose the same flexible system for delivering water. This approach was reinforced by the fact that, for a long period of time, the Egyptians in the Nile Valley seem to have used basic technology in their water installations.

It must be conceded that it is sometimes hard to find solid evidence with which to assess this topic for certain settlements.^22^ Nevertheless, from what we know, we can observe some degree of continuity in the model of water supply until the New Kingdom. From this time, however, the gradual development of wells and technological developments seem to have prompted slow changes to the system. The appearance of more elaborate wells in the valley seems to have modified the approach to water supply and also the role and the implication of the state in it. By gradually placing a source of water in the towns, close to the inhabitants who were now able to supply themselves directly, the state seems to have withdrawn slowly from one of its many responsibilities regarding the supply of provisions to the population.

## Footnotes

1 Preserved at the Ashmolean Museum, Oxford, E 3632. Discovered by Quibell (1900, p. 9, pl. XXVIc). For a recent study, see Gautier and Midant-Reynes (1995).

2 This article is a brief summary of some results obtained during my PhD thesis (see Driaux 2010), submitted to the Université Paris-Sorbonne (Paris IV), and now in preparation for publication.

3 Stela now preserved in Berlin (No. 17500), see Borchardt (1905). For the text and the translation, see Goedicke (1967, pp. 55–77).

4 Translation from Strudwick (2005, pp. 103–105).

5 For pyramid towns, see Kemp (2006, pp. 201–221).

6 On the definition of these installations, see Driaux (2010, pp. 234–254).

7 Note that neither Sarenput I nor Mentuweser have a specific title related to water and its supply. We have, moreover, no clear evidence that such a title existed.

8 These words will be studied in detail in a further publication.

9 For a study of the different words for “well”, see Franzmeier (2008) as well as Driaux (2010, pp. 234–254).

10 On the early 20th century excavations, see Peet and Woolley (1923, pp. 51–91, pl. XVI) and for more recent synthesis and fieldwork, see Kemp (1987).

11 On the excavations of this area, see Kemp (1984, pp. 60–80). For a short presentation and bibliographical references on *zîrs*, see Budka (2014, pp. 122–123).

12 On the village’s excavations, see in particular Bruyère (1939, pp. 1–78).

13 Abbreviation for “the great and noble Tomb of millions of years of Pharaoh on the west of Thebes”. See Cerný (2001, pp. 22–23).

14 On the chief workmen, see Cerný (2001, pp. 121–132), as well as for the scribes (pp. 191–230).

15 For a study of the documentation related to the water-carriers, see in particular Eichler (1990, 1991).

16 On the basis of texts given by the ostraca, some calculations about the quantity of water delivered have been made; a figure of 100 l of water, as a daily ration, per household has been estimated (see Helck 1965, pp. 845–847; Janssen 1979, pp. 12–13). In a settlement of at least 60 houses, that implies a huge quantity of water delivered each day and a large number of trips to the valley for the water-carriers. But, as mentioned by Janssen (p. 12), the meaning of some of these texts “is far from clear”. The documentation and its interpretation need perhaps to be reconsidered. See for example, Gentet and Sweydan (1994) and note that the hypothesis developed by the authors (p. 183) about the cost of the water deliveries for the administration might be more evidence for the concrete involvement of the state in the water supply.

17 For a brief presentation of these fortresses and their activities, see Kemp (2006, pp. 231–241).

18 From the first dynasties, and even earlier, during the Neolithic (see for instance the wells discovered in the Nabta Playa area, in the south-east part of the Western Desert, Kobusiewicz 2003) to the New Kingdom. On the wells and their evolution, see Driaux (2010, pp. 33–96).

19 The text stands on the north wall of the temple of Seti I at Wadi Kanais (the site is located 55 km east of Edfu). Comprising 14 lines of inscription it records the construction of the well and the temple during the ninth year of Seti’s reign. For the complete hieroglyphic text and translation, see Schott (1961, pp. 141–128, pl. 19); K*RI* I 65,14–67,11; K*RITA* I, pp. 56–58 and Davies (1997, pp. 208–213).

20 Translation from K*RITA* I, pp. 56–57, 65,17–66,11.

21 On the problems with the supply and delivery during the reign of Ramesses III and the strikes, see Valbelle (1985, pp. 190–193).

22 The author will publish additional evidence shortly.
